# Possible Prophylactic Approach for SARS-CoV-2 Infection by Combination of Melatonin, Vitamin C and Zinc in Animals

**DOI:** 10.3389/fvets.2020.585789

**Published:** 2020-12-03

**Authors:** Sabiha Fatima, Syed Shams Zaidi, Ashwag Saleh Alsharidah, Feda S. Aljaser, Naheed Banu

**Affiliations:** ^1^Department of Clinical Laboratory Sciences, College of Applied Medical Sciences, King Saud University, Riyadh, Saudi Arabia; ^2^Director of Pharmacy, Goulburn Valley Health, Shepparton, VIC, Australia; ^3^Department of Physiology, College of Medicine, Qassim University, Buraidah, Saudi Arabia; ^4^Chair of Medical and Molecular Genetics Research, Department of Clinical Laboratory Sciences, College of Applied Medical Sciences, King Saud University, Riyadh, Saudi Arabia; ^5^Department of Physical Therapy, College of Medical Rehabilitation, Qassim University, Buraidah, Saudi Arabia

**Keywords:** chronic immobilization stress, oxidative biomarkers, inflammation, melatonin, vitamin C, zinc

## Abstract

SARS-CoV-2, an epidemic, causes severe stress in both human and animals and may induce oxidative stress (OS) and increases susceptibility to infection. Domestic animals are found infected by their COVID-2 suffering owners. Chronic immobilization stress (CIS), a model of psychological and physical stress of confinement, can trigger depression and anxiety in animals. We evaluated the ameliorative effect of the proposed SARS-CoV-2 prophylactic drugs melatonin, vitamin C, and zinc on CIS-induced OS, inflammation, and DNA damage in rats. Forty male Swiss albino rats (200–250 g, 7–9 weeks old) were divided into five groups as controls, CIS, treated with melatonin (20 mg/kg), and vitamin C plus zinc [VitC+Zn (250 + 2.5 mg/kg)] alone or in combination (melatonin+VitC+zinc) subjected to CIS for 3 weeks. CIS was induced by immobilizing the whole body of the rats in wire mesh cages of their size with free movement of head. Exposure to CIS significantly compromised the circulatory activities of superoxide dismutase, catalase, and glutathione with enhanced malondialdehyde, inflammatory markers (IL-6, IL10, and TNFα), and lymphocyte DNA damage in comparison to controls. Treatment with melatonin and VitC+Zn alone or in combination significantly restored the altered biochemical parameters and DNA damage of stressed rats to their respective control values. However, the cumulative action of melatonin with VitC+Zn was more effective in alleviating the CIS-induced OS, inflammation, and DNA damage. The present study indicates that the antioxidant combination can be an effective preventive measure to combat severe psychological and confinement stress-induced biochemical changes in animals due to abnormal conditions such as SARS-CoV-2.

## Introduction

The epidemic of SARS-CoV-2 has induced severe psychological (social distancing) and confinement (physical) stress to prevent the spread of the disease. Stress has been implicated as a susceptible factor for oxidative stress (OS) induction, which compromises the immune system, influencing both human and animal health (1). SARS-CoV-2 infection causes up-regulation of systemic inflammation as shown by enhanced levels of pro-inflammatory cytokines interleukin 1 (IL-1), IL-6, and tumor necrosis factor (TNF) alpha, with higher concentrations of the anti-inflammatory cytokine IL-10. Further, it may result in an OS generation, characterized by reactive oxygen and reactive nitrogen species production, along with a concomitant deficiency of *in vivo* antioxidants. Enhanced OS induces inflammation and suppresses the immune response increasing the susceptibility to many diseases as well as incidences of viral infections in both animals and humans (2).

Psychological and confinement stress can lead to sleep deprivation, which is another factor that can negatively impact immune health (3, 4). Stress has been reported to negatively influence the synthesis of melatonin in the pineal organ, which is commonly known as sleep hormone (5). Psychological stress can affect the quality of sleep; sleeping problems themselves can become an added source of stress (6, 7). Disturbed sleep during stress increases respiratory oxygen intake and metabolic turnover requiring an increased demand of oxygen that promotes the excessive generation of the free oxygen radicals and oxidative damage to the cellular components in animals (8). Thus, OS with lack of sleep and decreased melatonin synthesis due to stress can weaken the immune system, increasing the vulnerability of animals and humans to viral infections such as SARS-CoV-2.

Based on the strong evidences on the role of OS, a balance of free radicals by exogenous antioxidants are the first lines of choice to maintain a proper physiological function in chronic restraint stress, that it is primarily a psychological stressor model. Melatonin hormone, which is secreted mainly at night to maintain sleep–wake rhythm, also has an endogenous antioxidant with anti-inflammatory properties that controls insomnia, reduces anxiety, and stimulates immunity (9, 10). Melatonin has been shown to relieve acute respiratory stress caused by viruses, bacteria, and radiation and can help decrease lung fibrosis, which is a significant complication of SARS-CoV-2 (11, 12). Bats, which were thought to be the primary cause of COVID-2 spread, have high levels of melatonin while they themselves do not suffer with the disease (13). Melatonin can also inhibit the SARS-CoV-2 activated inflammasomes like NLRP3, which triggers the sequence of cytokine storm that is responsible for extreme lung inflammation, injury, and acute respiratory distress syndrome (9, 12).

Vitamin C (VitC) and zinc (Zn) are the antioxidants acting as scavengers of free radicals, anti-inflammatory and immune-boosting micronutrients with an effect on stress reactivity (14–17). Zinc is important for immune function in production of antibody and white blood cells. Zinc deficiency is reported to suppress Nrf2 activity, which protects against OS by scavenging reactive oxygen and nitrogen species, as zinc regulates its expression and transcription (18). Supplementation of zinc enhanced the ability of polymorphonuclear cells to combat infection and vitamin C decreased the susceptibility to viral respiratory infections and pneumonia (19). Deficiencies of vitamin C and zinc severely depress the immune response and cause decreased resistance to infection (20). Moreover, both vitamin C and zinc are cofactors for several enzymes (21). Under normal conditions, domestic animals such as cat and dogs including ruminants can synthesize vitamin C; however, during stress and infection, vitamin C and Zn levels decline rapidly in plasma (22). Lower levels of VitC and Zn in human and animal models are associated with increased cortisol, a leading stress hormone synthesized after a psychological or physical stressor (23, 24). Further, it is reported that susceptibility to viral respiratory infections and pneumonia can be decreased by vitamin C (19). In addition, the replication of a variety of RNA viruses can be efficiently impaired by increased intracellular zinc concentration and zinc ionophores (25).

Though recently several review articles have been written and published on the preventive role of melatonin, zinc, and vitamin C on SARS-CoV-2, the experimental evidence are scarce. Based on the previous studies, we selected restraint stress model for both psychological stress of confinement (social distancing) and physical stress of immobility, which compromises both OS and inflammatory markers, and may mimic the present scenario. The study is designed to evaluate and compare the possible prophylactic efficacy of the proposed drugs melatonin and vitamin C plus zinc for SARS-CoV-2, alone or in combination in alleviating the OS, inflammatory markers, and DNA damage.

## Materials and Methods

Melatonin obtained from Sigma-Aldrich (St. Louis, MO, USA) was dissolved just before use in 1% ethanol and 99% normal saline. Vitamin C + zinc (VitC+Zn) tablets containing vitamin C 500 mg and zinc 5 mg (HealthvitC-Vitan-Z) was obtained from West Coast Pharmaceuticals Works Ltd (Ahmadabad, India). The other chemicals were procured from standard suppliers.

### Chronic Immobilization Stress (CIS) Protocol

Animals were subjected to CIS protocol as previously described (26). Briefly, stress was induced by immobilizing the rats by placing them individually in wire mesh cages of their sizes fixed to a wooden board, which prevented movement of their (whole body) trunks and limbs but allowed free movement of the head. Protocols used for the immobilization stress abide by the guidelines of the Animal Welfare Ethics Committee (CAMS 108-3839) of King Saud University, Riyadh. Animals were supplied with a standard diet (51.5% nitrogen free extractives, 19.2% crude proteins, 4.1% crude fat, 6.1% crude fiber, 5.8% crude ash, 11.3% moisture, and 2% vitamins and minerals) and water ad libitum throughout the experiment. However, food and water were withdrawn during the stress treatment.

### Experimental Design

Forty healthy male Swiss albino rats (200–250 g, 7–9 weeks old) were accustomed for 1 week to standard rat chow feed and water accessible ad libitum with interchanging light and dark cycles of 12 h and at 20–23°C temperature. Rats were divided into five groups of eight rats each. The treatment drugs were given by intraperitoneal injections. The doses were selected based on the pilot studies done in our laboratory (data not shown).

**Group I** (Control) Non-stressed control rats were handled every day for 10 min to nullify the non-specific handling effects and were given injections of normal saline intraperitoneally.

**Group II** (CIS-stress alone) Rats were subjected to CIS protocol for 3 h at 8 am for 3 weeks as discussed above.

**Group III** (Melatonin-treated stressed group) Rats were daily injected with melatonin (20 mg/kg body wt) at 8 pm and subjected to restraint stress at 8 am for 3 weeks same as group II.

**Group IV** (VitC+Zn**-**treated stressed group) Rats were daily injected with vitamin C+zinc (250 + 2.5 mg/kg body wt, respectively) in the morning and subjected to restraint stress at 8 am for 3 weeks same as group II.

**Group V** (VitC+Zn+melatonin-treated stressed group) Rats were daily injected with combined doses of MLT (10 mg/kg body wt) plus VitC+Zn (250 + 2.5 mg/kg body wt) and concurrently subjected to CIS for 3 weeks as group II.

All the rats were healthy during the experimental period and none died; no significant change in their body weight was recorded, though the weight of stress-treated rats was slightly decreased as compared to the other groups (data not given).

After 3 weeks of CIS paradigm, all the experimental animals were anesthetized with ether and blood was subsequently collected in heparinized tube from the retro-orbital sinus. Plasma from heparinized blood was separated by centrifugation at 3,000 rpm for 15 min at 4°C and stored at −80°C for further analysis of biochemical parameters.

### Biochemical Assay

#### Antioxidant Enzymes

The activity of superoxide dismutase (SOD) in plasma was assayed by monitoring the inhibition of auto-oxidation of 8 mM pyrogallol solution in 0.05 M tris succinate buffer, pH 8.2, every 30 s for initial 3 min at 420 nm with or without the enzyme protein. One enzyme unit is defined as the amount of enzyme required to cause 50% inhibition of the rate of pyrogallol auto-oxidation (27).

Plasma catalase (CAT) activity was determined in 50 mM potassium phosphate buffer (pH 7.4) by following the rate of decomposition of 19 mM hydrogen peroxide (H_2_O_2_). The decrease in the absorbance for H_2_O_2_ decomposition was recorded after every 30 s for 3 min at 240 nm (28). One unit of CAT activity is calculated as nanomoles of H_2_O_2_ consumed min^−1^ mg^−1^ protein.

### Total Reduced Glutathione (GSH)

Circulatory GSH was determined by the method of Jollow et al. (29). Briefly, 50 μl of plasma was added to 1 ml of 4% sulfosalicylic acid and allowed to stand for 5 min at 25°C and centrifuged at 4,000 rpm at 4°C for 15 min. In the supernatant, 0.1 M of potassium phosphate buffer (pH 7.4) and 0.01 M 5,5-dithiobis-2-nitrobenzoic acid (DTNB) was added. The yellow color developed by the reaction of GSH with DTNB was read at 412 nm, and the concentration of reduced GSH was determined as nmol/mg protein.

#### Lipid Peroxidation (LPO)

Lipid peroxidation in the sample was determined spectrophotometrically by the method of Beuge and Aust (30). Briefly, the samples were mixed with 0.8% thiobarbituric acid (TBA) and 15% trichloroacetic acid (TCA) and incubated at 95°C for 20 min to precipitate protein. After cooling, the mixture was centrifuged at 4,000 rpm at room temperature for 15 min. In the supernatant, the absorbance of pink chromogen formed by the MDA (a thiobarbituric acid reactive species: TBARS)–TBA complex was read at 532 nm against a reagent blank.

### Inflammatory Markers Assay

The ELISA for TNF-α, interleukin-6 (IL-6), and interleukin-10 (IL-10) in the plasma was done using rat TNF-α, IL-6, and IL-10 Quantikine ELISA assay kit (R&D Systems, Minneapolis, USA).

#### Protein Estimation

Protein concentrations were estimated by the method of Lowry et al. (31) using bovine serum albumin as standard.

### Lymphocyte Isolation for Comet Assay

Immediately after blood collection, lymphocytes were isolated using Histopaque 1077 from the heparinized blood diluted in PBS (Ca^2+^ and Mg^2+^ free) to measure DNA damage. The isolated cells (~2 × 10^5^) were finally suspended in RPMI 1640.

Single alkaline cell gel electrophoresis (comet assay) was performed to assess the DNA damage in the lymphocytes as described earlier (32). The assessment of cellular DNA damage was done by tail length (migration of DNA from the nucleus in mm) automatically generated by Komet 5.5 image analysis system.

### Statistical Analysis

Data expressed as group mean ± SEM of eight values were analyzed by one-way ANOVA followed by Tukey's *post-hoc* test for the comparison between the control or stressed and treated groups. All the statistical analysis was performed using GraphPad Prism7 (GraphPad Software Inc). Statistical significance was set at *P* < 0.05.

## Results

### Effect of MLT and VitC+Zn Treatment on CIS Generated OS and Inflammation

Twenty-one days of CIS significantly (*P* < 0.001) deranged the antioxidant defense system in the systemic circulation when compared to the control group. Lipid peroxidation measured in terms of the MDA level increased, whereas the GSH level decreased significantly (*P* < 0.001) on stress exposure. Moreover, stress treatment led to a significant (*P* < 0.001) drop in the activities of the antioxidant enzymes SOD and CAT in comparison to control group (Table 1).

**Table 1:** The effect of CIS induced alterations in the circulating enzymatic and non-enzymatic antioxidant parameters and their modulation by treatments with Melatonin (MLT) and Vitamin C plus Zinc (VitC+Zn) either alone and in combination.

**Groups**	**SOD (U/mg protein)**	**CAT (U/mg protein)**	**GSH (nmol/mg protein)**	**MDA (nmol/mg protein)**
Control	421.0 ± 7.8	18.7 ± 1.31	1.13 ± 0.01	10.41 ± 1.10
CIS	285.0 ± 5.41^*^	13.1 ± 1.01^*^	0.64 ± 0.01^*^	6.43 ± 1.01^*^
CIS+MLT	379.5 ± 6.12^**^	16.9 ± 1.12^**^	1.03 ± 0.03^**^	8.91 ± 1.05^**^
CIS+ (VitC+Zn)	388.7 ± 5.83^**^	17.0 ± 1.21^**^	0.95 ± 0.02^**^	8.72 ± 1.11^**^
CIS+MLT+(Vit C+Zn)	419.3 ± 6.71^#^	18.5 ± 0.98^#^	1.16 ± 0.02^#^	10.37 ± 0.95^#^

*Data represent the mean ± SEM (n = 8)*.

* *P < 0.001 as compared to control*.

** P < 0.01 and

# *P < 0.001 as compared to CIS*.

In addition to OS markers, exposure to CIS caused a significant increase (*P* < 0.01) of plasma levels of cytokines such as TNFα, IL-6, and IL-10 as equaled to the control group (Table 2). When MLT and VitC+Zn were given alone or in combination with melatonin, they significantly (*P* < 0.001) reinstated the altered OS parameters and inflammatory cytokine markers to their respective values of the CIS group.

**Table 2:** The effect of Melatonin (MLT) and Vitamin C plus Zinc (VitC+Zn) treatments alone and in combination on the circulatory TNF-α, IL-6, and IL-10 concentrations in rats exposed to chronic immobilization stress (CIS).

**Groups**	**TNFα pg/ml**	**IL-6 pg/ml**	**IL-10 pg/ml**
Control	25.4 ± 1.1	38.3 ± 1.3	41.1 ± 2.5
CIS	34.6 ± 1.3^*^	51.6 ± 1.5^*^	52.3 ± 1.9^*^
CIS+MLT	28.5 ± 0.97^**^	44.5 ± 1.3^**^	43.8 ± 2.1^**^
CIS+ VitC+Zn	29.2 ± 1.0^**^	43.7 ± 1.1^**^	44.3 ± 1.8^**^
CIS+MLT+VitC+Zn	26.2 ± 0.95^#^	37.8 ± 1.2^#^	40.4 ± 2.1^#^

*Data represent the mean ± SEM (n = 8)*.

* *P < 0.001 compared to control*;

** *P < 0.02 compared to CIS*;

# *P < 0.001 compared to CIS*.

### Effect of MLT and Vitamin C+Zinc Treatment on CIS-Induced DNA Damage in Lymphocytes

**Figure 1:**
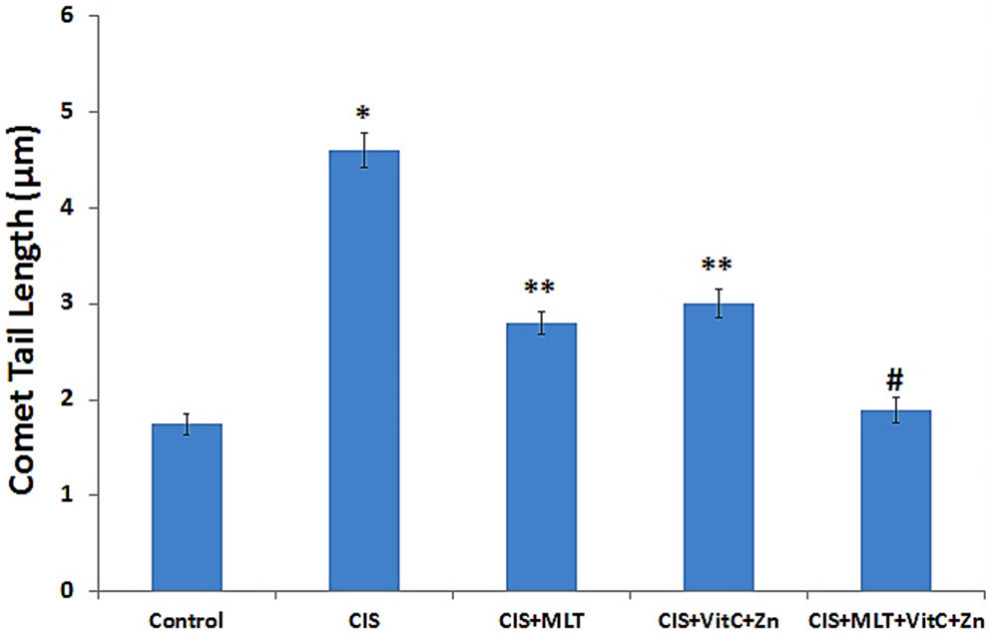
Chronic immobilization stress (CIS)-induced lymphocyte DNA damage measured as comet tail lengths. The simultaneous 21-day treatment with MLT and VitC+Zn alone or in combination significantly restored the CIS-induced lymphocyte DNA damage. *P < 0.001 compared to control; **P < 0.01 compared to CIS; ^#^*P* < 0.001 compared to CIS. Melatonin (MLT), vitamin C (VitC), and zinc (Zn).

Comet tail length, a measure of DNA damage, was found to be significantly (*P* < 0.001) increased by CIS in the lymphocytes, indicating substantial DNA damage as compared to the control group. However, MLT and VitC+Zn treatment singly or in combination along with CIS significantly (*P* < 0.001) reduced the degree of damage in the lymphocyte DNA as compared to the CIS group (Figure 1), but a reversion toward control values was observed.

The combinatorial dose of MLT with VitC+Zn in the presence of CIS was more effective in mitigating stress-stimulated alterations in the antioxidant status, inflammatory markers, and lymphocyte DNA damage in circulation when compared with the treatment of either MLT or VitC+Zn and stress alone. Moreover, no statistically significant variance was observed in the redox and inflammatory parameters and DNA damage in the groups treated with antioxidants alone or in combination (without CIS) compared to the control group (results not shown).

## Discussion

The current SARS-CoV-2 pandemic has developed fear, depression, and isolation with enhanced stress and sleep-related issues. The stress and anxiety can be equally felt by both the pets and their owners, developing signs of stress. Sleeping pattern is severely affected due to continuous stay at home; this may decrease the synthesis of melatonin, which is best synthesized during night sleep (13). Lack of exercise compromises the immune system too. This makes the individuals more susceptible to SARS-CoV-2 infection in an antioxidant and immune-compromised state. The abilities of vitamin C, a powerful water-soluble antioxidant, and zinc along with their antioxidant properties and diverse effect on immune system suggest their significant role in the prevention of stress-induced OS, depressed immunity, and viral infection (33–35). Further, melatonin's anti-inflammation, anti-oxidation, immune-enhancing, fibrosis-preventing actions on respiratory syncytial virus models and its potential role as sleep-promotion support a rationale for its use in viral diseases (13). Bats, the primary suspected source of SARS-CoV-2, are nocturnal animals having high levels of melatonin, which may be responsible for their high anti-viral resistance (36, 37). The spread of SARS-CoV-2 from animal to animal or human or vice versa is still controversial and a subject of investigation. The worldwide pandemic might have been caused by wildlife trade from Wuhan, China (38).

There lies a delicate balance between *in vivo* oxidant and antioxidant status. Any imbalance between OS and compromised antioxidant levels can alter cellular functions by modifying the macromolecules and increasing the susceptibility to tissue damage (39). An abnormal increase in OS is mainly due to the generation of ROS by mitochondria (40). In the present study, we observed that 21 days of CIS altered the OS parameters. The decreased level of endogenous antioxidant GSH, SOD, and CAT levels along with increased level of MDA, the marker for lipid peroxidation in the blood, indicates OS generation during immobilization stress, similar to our earlier observation (32). The diminished level of GSH, the major cytosolic reduced thiol, responsible for maintaining the redox balance and protecting the cell destruction from the harmful effects of lipid peroxides, may also be the cause of enhanced lipid peroxidation. Moreover, decrease in the activities of the primary antioxidant enzymes SOD and CAT, the first line of defense in the riddance of toxic free radicals and electrophiles, further contributed to the redox imbalance. Hence, during CIS, significant decrease in the activities of SOD and CAT could be due to ROS-induced enzyme/protein denaturation or their overutilization to scavenge the products of OS (41). Furthermore, the alterations in the structure and function of proteins and lipids and the accretion of free radicals in the nucleus and mitochondria can affect nucleic acids directly or indirectly (42). The enhanced lipid peroxidation and compromised activities of the antioxidant enzymes were also concomitant with increase in lymphocyte DNA damage (32). MDA, the most mutagenic product of lipid peroxidation, reacts with DNA to form adducts with guanosine and adenosine bases, which can cause strand breaks (43). Significant DNA damage as depicted by increased DNA tail length was detected in the lymphocytes of the CIS-treated rats. Incapability of the cells to repair DNA due to decreased antioxidant defense or oxidation of the cellular components including DNA could be the cause of genotoxicity in the lymphocytes of the stressed animals. Thus, the whole compromised state makes the individuals more susceptible to SARS-CoV-2 infection.

Both OS and free radicals play a significant role in the inception and progression of inflammation, a primary symptom seen in SARS-CoV-2 infection (44). OS has been shown to activate NF-kB, which regulates the expression of several genes responsible for the production of inflammatory cytokines (45). OS-induced inflammation in the present study is indicated by the increased concentration of TNF-α, a key inflammatory cytokine along with IL-6 and IL-10. GSH has been reported to play a key role in the control of pro-inflammatory processes in the lungs (46). Thus, depleted endogenous GSH could be the cause of inflammation in stressed rats. Although GSH prevents the production of most inflammatory cytokines, it is also needed to maintain an adequate interferon gamma production, which is essential for the host defense (47, 48). Cells maintain a high vitamin C concentration especially in leucocytes, eyes, the adrenal and pituitary glands, and the brain (49), while infection and stress reduces the levels of both vitamin C and zinc (14).

In general, increase in OS due to suppressed endogenous antioxidant defense system is mitigated by antioxidant supplements. Rats treated with melatonin and vitamin C plus Zn singly or in combination showed attenuation in altered biological markers of OS, inflammation, and DNA damage. Antioxidant treatment showed reduction in CIS-induced OS by diminishing lipid peroxidation and increasing the activities of enzymatic antioxidant, as well as glutathione, another very important non-enzymatic antioxidant which is important in suppressing inflammation. Further, melatonin improves the anti-oxidative defense within mitochondria by enhancing reduced glutathione and superoxide dismutase levels to inhibit peroxidation and infection generated due to OS (14, 50). The lethality of coronavirus was enhanced in antioxidant protection-deprived bats. Moreover, the deletion of ROS-generating machinery in mutated animals increased susceptibility to respiratory viral infection (51). Hence, the restoration of antioxidant status and inflammation by melatonin, vitamin C, plus Zn caused a decrease in DNA damage as compared to the corresponding stressed mice. However, the combinatorial treatment of melatonin, vitamin C, plus Zn was more effective than their alone doses in restoring the plasma antioxidant status, lipid peroxidation, inflammation, and lymphocyte DNA damage in the chronic psychological/physical stress-exposed animals.

Thus, previous findings and the result of the current study advocate that melatonin, vitamin C, and zinc supplementation can be an effective combination not only to combat the stress responses and depression but also to strengthen the immune system either as a prophylactic measure or supplemental therapy during the current SARS-CoV-2 pandemic.

## Conclusion

The combinatorial dose of melatonin, vitamin C, and zinc restored the OS-attenuated *in vivo* antioxidant, lymphocyte DNA damage, and immunological parameters significantly than the alone treatment. Thus, this combination can be used both as an effective prophylactic and as supplemental therapy during the current SARS-CoV-2 pandemic to combat stress and depression and to strengthen the immune system. This combination may also lessen the severity of the viral infection in both human and animal.

## Data Availability Statement

The raw data supporting the conclusions of this article will be made available by the authors, without undue reservation.

## Ethics Statement

The animal study was reviewed and approved by the Animal Welfare Ethics Committee of King Saud University, Riyadh, KSA. The approval of Animal Welfare Ethics Committee (CAMS 108-3839).

## Author Contributions

SF and NB: conceptualization, methodology, interpretation, and writing—review and editing. SZ: conceptualization and design. AA: formal analysis and revision. FA: critical reviewing and editing. All authors consented to publish this version of the manuscript. All authors contributed to the article and approved the submitted version.

## Conflict of Interest

The authors declare that the research was conducted in the absence of any commercial or financial relationships that could be construed as a potential conflict of interest.
